# Impairment, disability and fatigue in multiple sclerosis

**DOI:** 10.22088/cjim.9.3.244

**Published:** 2018

**Authors:** Shahnaz Shahrbanian, Pierre Duquette, Nancy E. Mayo

**Affiliations:** 1 School of Physical and Occupational Therapy, Faculty of Medicine, McGill University, Montreal, Canada; 2 Faculty of Sport Science, Bu-Ali Sina University, Hamedan, Iran; 3 Department of Physical Education, Faculty of Humanities, Tarbiat Modares University, Tehran, Iran; 4 Notre-Dame Hospital (CHUM), University of Montreal, Montreal, Canada; 5 Division of Clinical Epidemiology, McGill University Health Center, Royal Victoria Hospital, Montreal, Canada.

**Keywords:** Multiple sclerosis, Pain severity, Pain presence, Fatigue, MS-related disability

## Abstract

**Background:**

Identifying the predictors of pain is important for both health professionals and researchers, because pain has repeatedly been found to be a strong predictor of activity limitations and participation restrictions. The objective of this study was to determine the predictors of pain presence and severity in a large, well-designed sample of community dwelling individuals with multiple sclerosis (MS).

**Methods:**

This was a cross-sectional study. A center-stratified random sample including 188 persons with MS were recruited from three major MS clinics in the Greater Montreal, Canada. Main outcomes included pain prevalence and severity. Predictor variables included depression, anxiety, perceived health status, fatigue, sleep problems, and perceived cognitive deficits. Participants completed three questionnaires: the first asked about the socio-demographic and clinical information of the subjects, the second assessed the pain characteristics of the subjects, and the third covered the predictor variables.

**Results:**

The prevalence of pain in our sample was 42%. MS- related disability was found to be in the main predictor for both pain presence and intensity. Fatigue also was a main contributor to pain presence. The results of this study also showed that pain was associated with higher levels of depression, anxiety, sleep problems, and perceived cognitive deficits, and diminished perceived health status.

**Conclusions:**

The results of this study indicated that pain is a common symptom among people with MS. Pain presence was predicted by MS-related disability and fatigue, while pain intensity was only predicted by MS severity.

Multiple sclerosis (MS) is a chronic, inflammatory autoimmune demyelinating disease of the CNS. Pain is a frequent complaint among individuals with MS (1-6). The annual cost of MS has been estimated at 2.5 billion dollars in the US, and $502.3 million in Canada (6). The high prevalence of pain among persons with MS and the cost of MS pain would indicate that this is an important area of research in clinical management. There are several risk factors found to be associated with pain in people with MS such as older age, female sex, longer disease duration, and greater disease severity (5). While the relationship between pain and other factors in MS has been widely investigated, the relationship remains controversial and there is still inconsistency with respect to the important various clinical and personal factors (7). Identifying the predictors of pain is important for both health professional and researchers, because pain has repeatedly found to be a strong predictor of activity limitations and participation restrictions (2-4). Pain impacts on different aspects of individuals’ life. In comparison to MS people without pain and the general population, individuals with MS pain report poorer health-related quality of life (HRQL) (4), poorer overall mental and general health, more social role limitation, and more depressive symptoms (8-11). Moreover, nearly half of people with MS and pain report that pain interferes with their daily living activities (2) and sleep (12).

There are substantial gaps in the literature on pain in MS. Despite the high prevalence and significant impact, MS-related pain is still poorly understood and often under-emphasized because of its complexity and subjective nature. In addition, assessing pain is an essential component to rehabilitation, as it has been widely accepted that a first step in improving the treatment of pain is its adequate assessment.

 Available information of MS related pain often is limited by value because of methodological and analytical problems. For the most part, previous studies have looked at pain as a uni-dimensional health outcome or have focused on only few dimensions of pain (e.g. intensity and duration) in their analysis. 

A comprehensive and detailed assessment of pain, along with its impact and predictors and its most related mediator variables such as those that have been provided in this study, as well as interpretation of results using appropriate statistical methods in a large and well methodologically designed study could improve our understanding of its nature and mechanism, and in turn contribute to the development of more targeted approaches to enhance pain management. Therefore, the objective of this study was to determine predictors of pain presence and intensity in a large, well-designed sample of community dwelling individuals with MS. While the treatment of MS-related pain is challenging, knowledge of main contributors to pain can facilitate its prompt diagnosis and management.

## Methods

**Design: **This was a cross-sectional study where the data were collected at a specific point in time from patients with MS, living in the Greater Montreal area, Canada.

**Participants: **The target population for this study consisted of persons with a diagnosis of MS since 1995. Patients with any** c**linical types of MS included: relapsing remitting (RR), primary progressive (PP), secondary progressive (SP), progressive relapsing (PR), and clinically isolated syndrome (CIS). 

The available population was all men and women who had been diagnosed since 1995 and registered at the three major MS clinics in the Greater Montreal including Montreal Neurological Hospital (MNH), Centre Hospitalier de l’Universite´ de Montreal (CHUM), and Clinique Neuro Rive-Sud (CNRS). A center-stratified random sample of 550 individuals with MS was drawn, of which 364 were contacted. From those who were contacted, only the first 192 who responded were only included, due to the limited budget. Following exclusion of three people with incomplete data and one person who did not attend the evaluation session, 139 women and 49 men comprised the study sample (52 % of those contacted). No significant difference was found between responders (n=188) and non responders (n=176) on age, sex, MS severity, date of diagnosis, and duration of symptoms. As is usual in the epidemiology of MS (1, 8), the sample consisted of more women than men (the ratio 2.8:1). Participants who had a relapse in the preceding month, participants younger than 18 years old, people with severe cognitive impairments, and those with pre existing health conditions affecting functioning, such as mental illness, heart disease, rheumatoid arthritis, malignancy, renal failure, HIV/Aids, or liver failure were excluded.

*Measures* *:* ***All measures were chosen for the purpose of this study adequately representing the components of the underlying construct; their validity and reliability have been determined; and they have been used in MS researches in previous studies.***

**Socio-demographic characteristics:** Socio-demographic factors such as gender, age, smoking status, education level, and employment status were recorded on the day of testing using the socio-demographic questionnaire.

**Disease-related characteristics:** The clinical records and medical charts of each participant were consulted to obtain data on MS type, and years since MS diagnosis and symptoms onset. 

Participants also were asked to report if they used disease modifying therapy (DMT). The severity of neurological impairment was scored by neurologists using the Expanded Disability Status Scale (EDSS), the standard measure for classification of MS related disability, ranging from 0 (no disability) to 10 (maximum disability) (13).

**Pain characteristics**

**Pain prevalence: **Patients were asked “Are you currently experiencing any pain regardless of intensity and localization?” Pain prevalence was determined by calculating the proportion of participants who answered ‘yes’ to this question. Additional pain questions were only administered to persons with pain.

**Pain severity:** To measure average, the lowest and worst pain severity over the previous week as well as pain at the time of evaluation, we used 0–10 numeric rating scale (NRS), with 0 indicating ‘No pain’ and10 indicating ‘the most painful sensation imaginable’(14). NRS was also used to classify the participants as having no pain (score 0), mild pain (scores 1–4), moderate pain (scores 5- 6) and severe pain (scores 7–10) (15). Reliability, validity, and responsiveness of NRS have been documented (16, 17).

**Predictor variables**

**Fatigue:** Fatigue was measured using the 4-item vitality subscale (VIT) of the RAND-36, ranging from 0 to 100 with a higher score indicates greater energy/ lower level of fatigue (18). The vitality subscale of RAND-36 is part of the MS quality of life (MSQOL)-54 instrument which has been widely used in MS literature for assessing fatigue (19-21), and its psychometric properties have been provided (18). 

**Sleep disturbance:** To assess sleep disturbance, we used a specific sleep questionnaire created from Rasch Analysis of the Pittsburgh Sleep Quality Index (PSQI) (22) containing 4 items that assess factors affecting sleep quality during the previous month. Total score ranges from 0 to 8, with a higher score indicating worse sleep quality during the previous month. Patients were also asked if their pain has interfered with their sleep during the last month. 

**Perceived health status:** Perceived health status was measured using the Euro-QOL visual analogue scale (EQ-VAS) (23). Subjects were asked to rate their overall health on 0 to 100 VAS scale, with 0 showing the worst perceived health and 100 showing the best perceived health. 

**Psychological well-being: **The levels of psychological well-being of participants were measured using the hospital anxiety and depression scale (HADS) (24). HADS has 14 items, and the total score ranges between 0 and 21; higher scores indicate worse depression/ anxiety symptoms (24, 25). The HADS is a reliable and valid tool and has been used in a number of MS studies (26, 27).

**Perceived cognitive impairment:** Perceived cognitive impairment was assessed using the Perceived Deficits Questionnaire (PDQ) (28). The PDQ items assess frequency of difficulties with attention/concentration, memory, and planning/organization during the past month on a 5-point Likert scale. PDQ contains 20 items, each from 0 (never) to 4 (almost always) with a maximum total score of 80; higher scores indicate greater perceived cognitive impairment (28). The validity and reliability of PDQ in MS has been widely accepted (28-30).

**Ethical considerations:** Study protocol and procedures were approved by the ethics committee of each participating hospital, informed consent was obtained and signed by all subjects on the day of testing 

**Statistical analysis**

Descriptive statistics (e.g, mean, standard deviations, and frequency) were used to describe the sample and summarized data. Associations between all variables were assessed using Spearman and Pearson correlation coefficients for categorical and continuous variables, respectively. Those variables that showed a significant relation with response variables (pain presence and severity) were considered as potential predictors in the regression analysis. The main outcomes of regression analysis were pain presence and severity.

 As outcomes were not continuous variables, multiple logistic regressions were used to analyze data. We considered pain presence as a categorical variable i.e pain present or pain absent. So we ran a nominal logistic regression. For pain severity, we considered it as an ordinal variable (0 to 10), thus we ran an ordinal logistic regression. 

The personal and clinical characteristics of participants as well as the explanatory variables were included in the analysis. Using stepwise multiple regressions, each predictor variable was entered into the model, and retained or discarded based on their contribution to the overall model (statistical significance at the 0.05, beta estimate, odds ratio, and R squared). Sample size calculation was based on the rule of thumb for regression analysis that is a minimum of 10 participants per predictor variable (31). 

Considering that in our final regression equations, there were 9 predictors, a sample size of 188 participants would be suitable and adequate sample size for this study (21 persons per each variable). If there were participants with missing data, they were excluded from the analysis. Statistical significance was considered for p-values less than 0.05. Statistical analysis was performed using the Statistical Analysis Systems (SAS) Version 9.2.

## Results

**Socio- demographic and clinical characteristics of the sample: **Socio- demographic and clinical characteristics of the sample are presented in table 1. 

**Table 1:** Characteristics of study participants with a comparison of pain and pain free groups

**Variables**	**Total ** **(n=188 )**	**Pain group** **(n=78)**	**Pain free group** **(n=110)**	**P value** +
**Current age (** $\overset{\text{®}}{x}$ **± SD)**	43 ± 10	44 ± 10	42 ± 10	*0.6
**Gender, N (%)**				
Women	139(74)	66 (35)	73(39)	**0.04
Men	49(26)	15(8)	34(18)	
**Education, N (%)**				***0.4
Primary school	2(1)	1(0.5)	1(0.5)	
High school	41(22)	22(12)	19(10)	
College	56(30)	23(12)	33(18)	
University	85(46)	32(17)	53(29)	
None	1(0.5)	0	1(0.5)	
**Employment N (%) **				
Employed	119(64)	38(20)	81(44)	**0.0002
unemployed	64(35)	39(21)	25(14)	
**Smoking status, N (%)**				
Regularly	38(20)	20(11)	18(10)	**0.3
Irregularly	10(5)	5(3)	5(3)	
Non smoker	140(75)	56(30)	84(45)	
**Years since diagnosis (** $\overset{\text{®}}{x}$ **±SD)**	3±4	3±5	3±3.5	*0.9
**Years since symptom onset (** $\overset{\text{®}}{x}$ **± SD)**	9±5	9±5	9±5	*0.9
**Disability, EDSS (Median± SD)**	2.4±2	3±2	2±2	*0.0001
**DMT, N (%) **				*0.6
Yes	110(85)	47(36)	63(49)	
No	20(15)	10(7.5)	(7.5)	
**MS subtype, N (%) **				
Relapsing-Remitting	97(78)	43(35)	54(43)	***0.03
Secondary progressive	7(5)	4(3)	3(2)	
Primary progressive	8(7)	2(2)	6(5)	
Primary relapsing	3(3)	2(2)	1(1)	
Clinically isolated syndrome	9(7)	0	9(7)	
**Pain impact**				
Sleep disorders (PSQI: $\overset{\text{®}}{x}$± SD)	6.5±1.5	6.7±1.5	7.4±1.6	*0.4
Perceived health status (EQ-VAS: $\overset{\text{®}}{x}$± SD)	73±17	66±19	78±13	*.0001
Fatigue (VIT- RAND-36: $\overset{\text{®}}{x}$± SD)	49.5±20	41±20	56±19	*.0001
Cognitive impairment (PDQ: $\overset{\text{®}}{x}$± SD)	24±15	29±14	20±14	*.0001
Depression (HADS: $\overset{\text{®}}{x}$± SD)	4±4	5.3±4	3.4±4	*0.001
Anxiety (HADS: $\overset{\text{®}}{x}$± SD)	5±4	6±4	4.6±3.6	*0.008

N: number

DMT: disease modifying therapy

*PSQI: Pittsburgh Sleep QualityIndex*

PFI: physical function subscale of RAND-36

EQVAS: EuroQol visual analog scale

VITA: RAND-36 Vitality scale of RAND-36

PDQ: Perceived Deficits Questionnaire

HADS: Hospital Anxiety and Depression Scale

+ The p-values given in the last column represent the difference between the 'pain group' and 'pain free group'

* T-test;

** Chi square;

*** Fisher test

**Pain characteristics of the sample: **Of the 188 persons, 42% identified pain as a symptom, and among those, 42% reported to have clinically significant pain (severity ≥4) at the time of evaluation. The mean value for rating of current pain at the time of evaluation was 3.3±2.3; the mean of lowest pain severity was 2.2±2; the worst pain severity was 6.8±2; and the pain average was 5.0±2. 40% of participants with pain reported that pain interfered with their sleep. In addition, participants without pain were more employed and reported greater energy level (lower fatigue), and daily living activity in comparison to participants with pain (p<0.05). Participants without pain also tended to show less perceived cognitive impairments, depression, and anxiety (p<0.05) (table 1). Regarding sociodemographic and clinical characteristics, there was no difference between the participants with and without pain on age, education, and smoking status, DMT, and duration of symptoms onset and diagnosis (p>0.05). However, the pain group showed a higher women-to-men sex ratio (4:1 vs. 2:1 in pain group), and higher EDSS scores (table 1).

**Factors associated with presence and severity of pain in MS: **The results of correlation analysis showed a statistically significant correlation between pain presence with gender, employment status, MS type, MS disability, fatigue, depression, anxiety, perceived health status, and perceived cognitive deficit (r = 0.1, r = -0.3, r = 0.2, r = 0.2, r = -0.34, r =0.2, r = 0.17, r = - 0.35, r = 0.3, p<0.05, respectively). MS- related disability, depression, and perceived health status also showed a statistically significant correlation with pain severity (r= 0.4, r= 0.3, r= -0.34, p<0.05, respectively). Nonetheless, no associations were observed in our study between pain severity with anxiety and perceived cognitive deficit. Neither pain presence, nor pain severity were associated with level of education, age, use of DMT, and years from symptom onset and diagnosis.

Table 2 displays the results of regression analysis for response variables. The results of nominal logistic regression analysis for pain presence showed that only fatigue and MS-related disability made a signiﬁcant contribution to prediction. Furthermore, analysis of maximum likelihood showed that for every unit change in fatigue score (RAND-36, lower score means less vitality so more fatigue), the probability of pain presence decreases by 0.96 (p=0.0001). MS-related disability made also significant contribution to prediction as for a unit increase in MS severity, the probability of pain presence increased by 1.2 (p=0.03). The results of ordinal logistic regression on pain severity also indicated that only MS-related disability had a significant effect on pain severity (p=0.001). This means that for every unit increase in MS severity, (EDSS score, higher score is worse), the probability of experiencing more severe pain increases by 1.4.

**Table 2:** Logistic regression model for pain presence and severity

**Parameter**	**Parameter estimate**	**Standard coefficient** *	**P-value**	**Odds ratio**
**Pain presence**				
Fatigue (RAND-36)	-0.03	-0.6	0.0001	0.96
MS-related disability (EDSS)	0.2	0.4	0.03	1.2
**Pain severity **				
MS-related disability (EDSS)	0.4	0.8	0.001	1.4

* Standardized coefficient = Parameter estimate x 1 Standard Deviation of each predictor

## Discussion

The purpose of the present study was to determine the contributors to pain intensity and presence among people with MS. MS-related disability was found to be the main predictor for both pain presence and intensity. Fatigue also was a main contributor to pain presence. The results of comparisons between individuals with MS and pain and those who were pain-free showed that pain was associated with higher levels of depression, anxiety, sleep problems and cognitive deficit, and lower levels of general health 

perception, and ability to work. The prevalence of pain in our sample was 42%. In addition, participants’ ratings of their worst pain intensity showed that 60% of those patients with pain reported severe pain (7–10 out of 10). This finding indicates that despite low prevalence of pain, pain severity was high in our sample, therefore reinforcing the need to identify pain reasons and looking for an effective approach to treat it adequately. There was no significant difference in age between participants with pain those and without pain. Regression analysis also revealed that neither pain presence, nor pain severity was associated with age. 

 These findings are consistent with the results of several studies (3, 4, 8), and are in contrast with the results of Hadjimichael (12), and Svendsen (1). Additionally, our results show that gender was correlated neither with pain severity nor with the presence of pain. These findings are similar to Douglas (5), and are in contrast to Kalia (4). Moreover, the results of regression analysis revealed that neither the duration of diagnosis nor symptom onset has been found to be associated with either pain presence or severity except for MS severity. These findings suggest that pain cannot be predicted solely based on the disease or personal characteristics and other factors play an important role. In accord with findings reported by previous studies (2, 3, 12) results of our regression analysis revealed that MS-related disability (measured by EDSS) was an important predictor for both pain presence and severity confirming that patients with greater disability are more likely to experience pain. Furthermore, similar to a previous study (2, 3, 8), we found that persons with pain were more likely to have greater MS disability than those without pain. 

Fatigue was found to contribute to pain presence in this study. This confirms the role of fatigue as the most disabling symptom of MS. In addition, these symptoms are possibly correlated through common etiology due to the simultaneous damage to nerve fibers across different parts of the CNS (32). Cognitive behavioral therapy (3), physical activity, rehabilitation programs, and energy conservation strategies have been shown to improve MS fatigue (34).

Similar to results reported before (2-4, 8), patients with pain tended to be more depressed and anxious than those without pain. Nevertheless, our results did not show any predictor effect of depression on pain severity and presence. This can be partly related to the fact that our sample reported no serious depression symptom. The mean depression scores of our sample were 4 out of 21 on HADS; cut off point is 8. Additional research is needed to understand whether additional unique factors may mediate this relationship in individuals with MS.

In agreement with Douglas (5), our results further revealed that persons reported more perceived cognitive deficit in the presence of pain. As Douglas (5) believes, this association can probably be related to the patients’ inabilities in coping strategies and problem-solving skills. Our finding further suggested that self-perceived health status could not act as a significant predictor for either pain severity or presence. Although, in agreement with other studies (2, 3), our results showed that participants with pain in comparison to those without pain were considerably more likely to report lower perceived health status. These findings encourage the implementation of specific approaches aimed at improving the self-perceived health status in people with MS. 

The current study has several strong points. It assessed a variety of MS symptoms using standardized measures which are used in MS population. Also, the study sample was randomly selected from 3 different clinics in Montreal from populations who were culturally diverse and living in different areas of the city. Besides, the sample included the whole range of disease severity, and type, consistent with a clinical spectrum of MS, so it could be representative of the general MS population. A further strength of this study was that, the present sample also included many men, thus providing a unique opportunity to study MS and pain in both genders, whereas many studies on MS and pain have included only a few men participants.

On the other hand, this study had several limitations. First, this was a cross-sectional study where subjects were assessed at one point in time, thus, the results do not show any cause and effect relation. Second, we purposely sampled individuals diagnosed after 1995. This was the year that DMT and magnetic resonance imaging (MRI) speeded the diagnosis and management of MS (35). Thus the results may not be generalized to all MS people who were diagnosed earlier. Third, as pain is a subjective experience, the scores could be subject to memory distortion, recall bias, and response shift. Finally, the fact that MS-related disability is a significant predictor of the presence and severity of pain invites caution when interpreting the associations between pain and employment status, perceived cognitive deficits, depression, anxiety, and perceived health status; all of these parameters are greatly influenced by MS-related disability. The results of this study help us to better predict the experience of pain among people with MS. Pain has repeatedly been found to be a strong predictor of activity limitation and participation restriction. 

The comparisons between participants with and without pain on job status in the current study also revealed an increasing proportion of participants not being employed in the presence of pain (63%). As MS is a disease that often affects young adults during their productivity years, this emphasizes the importance of early identification and treatment of pain. The identification of factors that diminish or trigger pain is important for clinicians, since it facilitates the development of targeted rehabilitative intervention to reduce pain. Research studies that compare the effects of pain on functioning in comparison to other MS symptoms are also necessary as their results would help clinicians to choose the priorities of treating these symptoms in persons with MS.

In conclusion** t**he results of the current study indicate that pain is a common symptom among people with MS. Pain presence was predicted by MS- related disability and fatigue, while pain severity was mainly predicted by MS disability. The considerable differences between participants with pain and those without pain on physical and psychological functions highlight the importance of accurate assessment and adequate intervention to manage pain in people with MS.
